# Informed Consent: How Much Awareness Is There?

**DOI:** 10.1371/journal.pone.0110139

**Published:** 2014-10-16

**Authors:** Daniel Purcaru, Adrian Preda, Daniela Popa, Marius Alexandru Moga, Liliana Rogozea

**Affiliations:** 1 Department of Medical Ethics, Transilvania University, Brasov, Romania; 2 Department of Clinical Research, Neomed Medical Center, Brasov, Romania; 3 Department of Psychiatry and Human Behavior, University of California Irvine School of Medicine, Irvine, California, United States of America; 4 University of California Irvine Neuropsychiatric Center, Orange, California, United States of America; 5 Faculty of Psychology and Sciences of Education, Transilvania University, Brasov, Romania; 6 Department of Medical and Surgical Specialties, Faculty of Medicine, Transilvania University, Brasov, Romania; 7 Department of Fundamental Disciplines and Clinical Prevention, Faculty of Medicine, Transilvania University, Brasov, Romania; Cardiff University, United Kingdom

## Abstract

Improving the informed consent process in clinical research is of constant concern to regulatory authorities in the field and presents a challenge for both the specialists and patients involved. Informed consent is a process that should adequately match the complexity of clinical research. In analyzing the behaviour of 68 patients during the informed consent process related to the clinical research performed at Neomed Clinical Center in Brasov, we found that many patients do not ask any questions (35.3%). From those who do, part of the questions (20,6%) referred to general aspects (addressed the form but not the gist) of the clinical trial, some (72,8%) referred to specific aspects of the clinical trial they will attend and others (6,6%) unrelated to the clinical trial. These results suggest a lack of interest, awareness, and understanding of the information presented in the informed consent form. The possible underlying causes of this attitude and its bureaucratic, ethic, and legal implications are discussed.

## Introduction

Advances in the design and targets of clinical research should translate to changes in the informed consent process that would guarantee that subjects always understand the potential costs and benefits of participating in a clinical research project. [1] Disclosing information to the patient based on the consent document does not assure that the patient fully understands what participation in a clinical research project involves. [2] The ever-increasing complexity of clinical research has created a more complicated informed consent process, and, at times, a confusing process that exceeds the subject's capacity to understand what participation entails. Participants may frequently not understand information disclosed to them in the informed consent process. [3].

Clinical trial participation offers subjects the promise to meet two of their most important expectations: an improvement, or even a cure, for their condition, and minimal costs associated with their treatment (possibly even free treatment). An improvement in the general condition of the patients during the study, even marked by brief episodes of mild adverse reactions, mask the procedural shortcomings in obtaining informed consent full awareness. The lack of the patient's awareness in giving consent could manifest itself when participants experience a decline in their general condition due to the development of the disease, associated conditions, or adverse reactions. Awareness is also important in long-term effects of the treatment, even if the patient's condition has improved, as adverse reactions could appear across shorter or longer period of time. [4].

The informed consent process begins with a conversation between the clinical investigator and the subject, who is presented with the option to participate in the clinical trial as well as an informed consent document. Subsequently, the subject may decide to either take the document home to study it alone or with their family, or review it at the study site. In both cases, the process continues with offering explanations and providing appropriate answers to the subjects’ possible questions, so that an educated and informed decision can be made. [5] Clinical investigators should be aware that the informed consent document alone does not assure the subject's full understanding of their participation. [6] Therefore, before the subject makes a decision, the research team should discuss the study purpose and procedures, risks and benefits, and the rights and obligations of the participant. [7] If the subject decides to participate, they will then sign the consent form. Both the subject and investigator receive a copy of the document. However, even after the subject decides to participate in the study, it is a best practice for the research team to continue to bring to their attention any new information that could affect their situation. [8] Before, during, or after the study, subjects should also have the opportunity to ask questions and discuss any issues they may have. Thus, informed consent is a continuous process, rather than a single training session. [9].

### Behavioural Analysis of the Patients during Informed Consent

We hypothesized that the likelihood of potential participants in a clinical trial of whether to ask questions or not, as well as their quality, is a reflection of the degree of their interest, awareness, and understanding of the information presented in the informed consent form. Consequently, we studied the subjects' behaviour before they signed the informed consent form.

Following an outpatient medical consult, the indication to participate to an ongoing clinical trial was established by the physician. On this occasion, the patients were invited to participate in that trial. If interested, they received an information brochure related to the trial. Preliminary discussions with the physician and questions & answers sessions before reading the brochure did not occur in the case of patients included in this prospective transverse observational study. The patients read the brochure without being offered any explanations at this point. The informed consent process allowed the patient to take the document home and study it alone or with the family, but this analysis included only patients who went through the informed consent process at the medical center in order to have the same conditions for all participants. Informing sessions consisted in reading the brochure, asking questions, providing answers, then explanations and discussions with the investigator, followed by another questions & answers session, if needed. These informing sessions lasted between an hour and an hour and a half, depending on the clinical trial described and the participant's reading skills. The decision to participate was made at that moment, or later.

**Figure 1:**
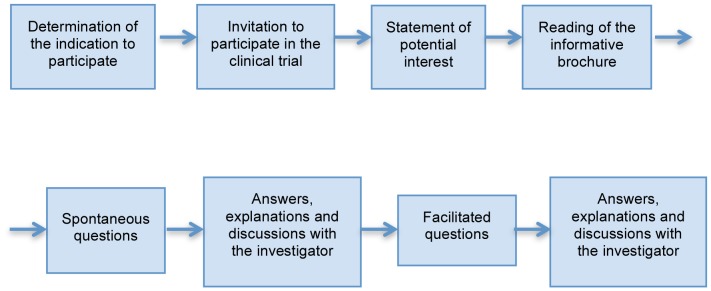
Layout of the analyzed informed consent process.

Our study did not involve direct research on subjects. The observation protocol requested the members of the research team to write down on a form all the questions asked by the patients. All the patients' questions, as well as the multiple questions asked by the same patient, were grouped according to the inquiry moment (Figure 1), as following:

- Spontaneous questions (after reading the brochure and before the discussion with the investigator)
- Facilitated questions (asked during or after the discussion with the investigator)

In order not to influence the participants' behaviour in this observational study, they were informed only at the end of the informed consent process that the investigator watched certain aspects of their attitude: whether the subjects asked questions, what kind and how many. The data were analyzed anonymously. Demographical data were collected, but not personally identifiable. We have not evaluated clinical data for our analysis. 68 patients with indication to participate in one of the 4 clinical trials (diabetes, hypertension, dyslipidemia, peripheral venous thrombosis) that took place at the Neomed Medical Center from Brasov between March and July 2013 gave their verbal consent to have these data processed. The local ethics committee of Neomed Medical Center specifically waived the need for written informed consent and also gave their approval for our analyses.

**Figure 2:**
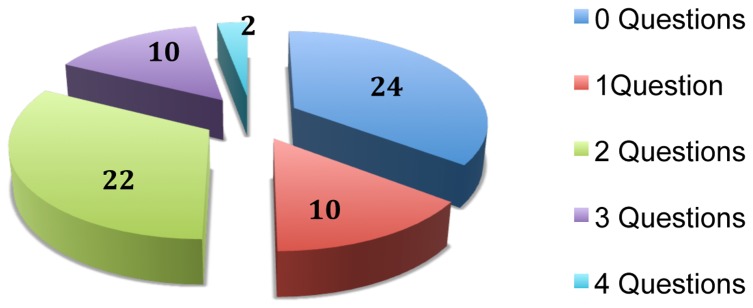
Distribution of the patients by the number of asked questions.

Therefore, as far as the number of questions addressed is concerned, out of the 68 forms submitted by the investigators who wrote down the received questions, most of the patients (24) did not ask any questions while the rest asked between 1 and 4 (Figure 2).

Regarding the type of asked questions, all 92 questions were grouped in 3 categories (Table 1):

**Table 1:** The distribution of the questions’ relevance.

92 Questions Asked:							
19 Questions regarding the general aspects of clinical research							
	2	What is a clinical trial					
	5	Previous research on human subjects					
	8	The costs associated with the participation at the clinical trial					
	4	The availability of the drug on the market					
67 Questions regarding the specific aspects of clinical trial							
	4	The frequency of visits at the medical center					
	4	The length of the visits at the medical center					
	10	The length of the treatment/clinical trial					
	4	Effectiveness of the study medication					
	16	Adverse reactions (AR)					
	4	Frequency of AR					
	7	The type/description of AR					
	5	The severity of AR and ways to solve them					
	14	The number and quantity of biological samples					
	5	Genetic tests					
	6	Discontinuation of current/concomitant medication					
	4	The effects of omitting the administration of a dose of research medication					
6 Questions unrelated to the clinical trial							
	2	Risk factors associated with the current condition					
	2	The evolution of the condition					
	1	Treatment after the end of the clinical trial					
	1	Lifestyle

- General aspects of clinical trials (19 questions) (e.g. what is a clinical trial, the availability of the study drug on the market, previous studies on human subjects, costs associated with participation)
- Specific questions related to the clinical trial (67 questions) (e.g. frequency and duration of the visits to the medical center, duration of the study, adverse reactions, placebo control, efficacy of the study drug, procedures)
- Questions unrelated to the clinical trial, regarding the disorder and its prognosis (6 questions) (e.g. lifestyle, associated risk factors, evolution of the disorder)

Following the descriptive statistical analysis of the data, we notice that out of all the subjects’ questions, addressed before and after the discussion with the investigator, the number of questions related to specific aspects of the clinical trial is predominant. Table 2 shows that the number of specific questions increases in higher education levels (60% for general education to 70% for high school studies and to 86% for university studies). Also, we noticed a decrease in the number of questions unrelated to the clinical trial (12% for general studies to 6% for high school studies to 0% for university studies).

**Table 2:** The distribution of the questions related to the graduated school.

Graduated school	No. of questions *before*the discussion		No. of questions *after*the discussion		No. of totalquestions	Total of GENERALquestions		Total of SPECIFICquestions		Total of UNRELATEDquestions		No. of totalquestions
General school	14	56%	11	44%	25	7	28%	15	60%	3	12%	25
High school	26	49%	27	51%	53	10	19%	40	75%	3	6%	53
University	8	57%	6	43%	14	2	14%	12	86%	0	0%	14

We wanted to find out if this predominance was statistically significant. To test if the awareness level of the patients’ informed consent varies according to the number and type of asked questions, the moment of being addressed (before and after the discussion with the investigator), as well as the educational level, we applied the ***χ^2^*** test for association. As it can be noticed in Table 3, we obtained significant differences in the ratio between the total number of questions and the number of specific questions in all groups of subjects, at both analysis moments (before and after the discussions with the investigator). Also, the values of the obtained Phi coefficient shows us that the relation between variables is strong, so we can state that there is a strong trend in the studied group to ask specific questions, compared to general and unrelated questions.

**Table 3:** The distribution of the questions' category related to the graduated school.

Questions	Test	General studies			High school studies			University studies		
		Value	df	Asymp. Sig. (2-sided)	Value	df	Asymp. Sig. (2-sided)	Value	df	Asymp. Sig. (2-sided)
Total of GENERAL* questions before discussion	Pearson Chi-Square	2,366667	4	0,669	6,073	4	0,194	6,073	4	0,194
	Phi	0,344		0,669	0,4			0,4		
Total of GENERAL* questions after discussion	Pearson Chi-Square	7,192308	4	0,126	9,585	6	0,143	9,585	6	0,143
	Phi	0,6		0,126	0,502			0,502		
Total of SPECIFIC* questions before discussion	Pearson Chi-Square	19,028	6	0,004	12,257	6	0,05	12,257	6	0,05
	Phi	0,975		0,004	0,56			0,56		
Total of SPECIFIC* questions after discussion	Pearson Chi-Square	15,87	6	0,014	22,504	9	0,007	22,504	9	0,007
	Phi	0,891		0,014	0,77			0,77		
Total of UNRELATED* questions before discussion	Pearson Chi-Square	0,523	2	0,77	7,917	4	0,095	7,917	4	0,095
	Phi	0,162		0,77	0,456			0,456		
Total of UNRELATED* questions after discussion	Pearson Chi-Square	6,928	2	0,031	7,374	6	0,288	7,374	6	0,288
	Phi	0,589		0,031						

Specific to the general studies group is the existence of a significant difference in the number of questions ratio, χ^2^ (2) = 6,928, p = 0,003. This relation shows that besides the specific questions, the subjects with general studies tend to ask questions unrelated to the clinical trial after the discussion with the investigator. The effect size indicator, Phi = 0,589, p = 0,003, shows that the relation between the two variables is strong.

We have observed that many patients do not ask any questions (35.3%). From those who do, part of the questions (20,6%) referred to general aspects (addressed the form but not the gist) of the clinical trial, some (72,8%) referred to specific aspects of the clinical trial they will attend and others (6,6) unrelated to the clinical trial. This attitude can reflect the lack of or insufficient awareness of the informed consent process.

## Discussion

There are a few possible explanations for our findings. First, presumably “a misunderstanding of the terms in the consent document may leave patients incapable of asking questions” [10], and “a scientific misinterpretation of a concept could create faulty assumptions”. [11] Even if the language used to disclose study information, oral or written, follows regulations (to be both non-technical and practical, in other words, intelligible for the patient) [12], in current practice, “the language of medical research contains one of the most specialized and technical terminologies”. [13] For instance, in common colloquial usage, the word “random” might suggest a chaotic, disordered situation. However, in clinical research terminology, “randomization” refers to the subjects’ selection, which minimizes the impact of confounders. Not surprisingly, previous studies report that revising the informed consent document did not improve patients' understanding of biomedical research. [14]–[16] There is skepticism about “improving subjects’ understanding of the clinical research process by changing the informed consent language (text)” [17], as even if “the efforts for its improvement are constant, few elements could actually be changed”. [18] We propose that the informed consent process can be improved by changing the process of obtaining consent instead of changing the informed consent content.

Another possible explanation is that subjects might have felt overwhelmed and intimidated by the quantity of information presented during the informed consent process. While the document is being read by a physician, the subject might lose attention; alternatively, when the subjects read the consent on their own, they might do so superficially, skimming the text as they would do with a utility contract. After the interviews with the research teams, we observed another situation that is unfortunately common: some of our patients did not appear motivated to understand the informed consent; they adhered to the study schedule and procedures, but they were not interested in the clinical trial or its objectives. For these patients, what mattered was the end result of the trial (an improvement in their condition), even if they were informed that this was not guaranteed. Lack of interest or reactivity (the fact that they don't ask questions) could also be due to cultural and educational conditions, which could be specific to Romania, as, historically, questioning was seen as a sign of insubordination and challenging authority. This attitude discouraged an open attitude and feedback from the individual due to the collective system that put people in their place, patronized them, and discouraged questioning. These factors may have prevented subjects from asking questions even if they were motivated and interested in learning more. As a result, subjects may be less informed than desired at the completion of the informed consent process.

### Bureaucratic, ethical, and legal aspects

From an administrative perspective, signing the informed consent document is proof that the subject understood the information presented and that they had authorized the investigator to proceed with the proposed study. Signing this document, however, does not demonstrate that the subjects actually read, understood, and retained this information. Over the last 10 years of practice in clinical research, the informed consent document has evolved from a short document to a longer one, sometimes exceeding 20 pages, containing a large quantity of complex information that describes many procedures that may be difficult to understand. Many pieces of information might be required to be presented from a liability perspective, but are irrelevant to the patient's consent. It is then the investigator’s responsibility to translate this complex information into simpler explanations and support the subjects in discussing the medical and research procedures.

From an ethical perspective, it is essential that the subjects understand what their participation in clinical research entails. The goal of the consent process is achieved only when patients understand the information disclosed in the informed consent and by the medical team. In current research practice, a number of patients who are less educated or older find that reviewing the informed consent document is quite tiring. In a culture where the patient-physician relationship is often dominated by deferring to the physician’s authority, subjects who do not clearly grasp the difference between a treating physician (seeing them as part of their clinical practice) and a clinical investigator (seeing them as part of their clinical research practice) often make statements such as, “doctor, you know better what I should do,” which is indicative of the expectation that the investigator (often times their physician) should function as a substitute decision maker. In this respect, patients may give their consent to participate in research because their physician tells them that they should. Also, “the real power scale in the patient-physician is out of balance, always tilted towards the physician” [19], and this state frequently results in inhibiting the patient. Under these conditions, making a collective decision is problematic and leaves subjects susceptible to manipulation. All of these factors, taken together with the time constraints that are usually present in the patient-physician relationship, can put the physician in the position of choosing the easy way to accept the “passing on” of the patient's consent. In such cases, it is the investigator’s responsibility to reject “passing on” the patient's consent, since it represents a major deviation from the GCP guidelines. This problematic situation requires more than just the clinician investigator providing extra time to offer every patient with a thorough explanation. The investigator must first also recognize the misunderstandings and doubts of the patient. Out of the 5 essential elements of an ethically valid informed consent (volunteering, capability, disclosure, understanding, and decision), “understanding is the most difficult to achieve”. [20] The most important part of a clinical trial related informed consent process is obtaining awareness of the information disclosed; this is not sufficiently demonstrated by signing the informed consent form, and rather needs to be confirmed by establishing a sufficient level of understanding.

From a legal perspective, there may be situations in which “both the investigators and the patients' lawyers need to unquestionably prove that a person has been informed and has understood what his voluntary participation in a clinical trial involves”. [21] Even if, legally, failure or lack of understanding does not lead to the invalidation of the consent, ethically, in the patient's best interest, we must use a method which would assure us that we have obtained the appropriate level of awareness by the informed consent process.
